# LncRNA HOTTIP leads to osteoarthritis progression via regulating miR-663a/ Fyn-related kinase axis

**DOI:** 10.1186/s12891-020-03861-7

**Published:** 2021-01-12

**Authors:** Xianwei He, Kun Gao, Shuaihua Lu, Rongbo Wu

**Affiliations:** 1 Department of Orthopaedics, Fudan University Jinshan Hospital, No.1508 Longhang Road, Jinshan District, Shanghai City, 201508 China; 2 Ze Tian Xing Zhi Di Cosmetology Clinic, Shanghai, 200000 China

**Keywords:** Osteoarthritis, Chondrocytes, FRK, miR-663a, lncRNAs HOTTIP

## Abstract

**Background:**

Long non-coding RNA (lncRNA) has been implicated in the progression of osteoarthritis (OA). This study was aimed to explore the role and molecular mechanism of lncRNA HOXA terminal transcriptional RNA (HOTTIP) in the development of OA.

**Methods:**

The expression of HOTTIP, miR-663a and Fyn-related kinase (FRK) in the OA articular cartilage and OA chondrocyte model induced by IL-1β was determined by qRT-PCR. CCK-8, colony formation and flow cytometry were used to determine the cell proliferation and apoptosis of OA chondrocytes. The specific molecular mechanism of HOTTIP in OA chondrocytes was determined by dual luciferase reporter assay, qRT-PCR, western blotting and RNA pull-down.

**Results:**

The expression of HOTTIP and FRK were up-regulated, while miR-663a was down-regulated in OA cartilage tissues. Knockdown of HOTTIP decreased the proliferation and induced the apoptosis of OA cartilage model cells, while overexpression of HOTTIP increased the proliferation and reduced the apoptosis of OA cartilage model cells. Moreover, HOTTIP could bind to miR-663a as competitive endogenous RNA. Inhibition of miR-663a expression could alleviate the effect of HOTTIP knockdown on the proliferation and apoptosis of OA cartilage model cells. Furthermore, FRK was found to be a direct target of miR-663a, which could markedly down-regulate the expression of FRK in OA chondrocytes, while HOTTIP could remarkably up-regulate the expression of FRK. In addition, miR-663a inhibition increased the proliferation and reduced the apoptosis of OA cells, while FRK knockdown reversed the effect of miR-663a inhibition on the proliferation and apoptosis of OA cells. Meanwhile, overexpression of miR-663a decreased the proliferation and induced the apoptosis of OA cells, while overexpression of FRK reversed the effect of miR-663a overexpression on the proliferation and apoptosis of OA cells.

**Conclusion:**

HOTTIP was involved in the proliferation and apoptosis of OA chondrocytes via miR-663a/ FRK axis, and HOTTIP/miR-663a/FRK might be a potential target for the treatment of OA.

## Background

Osteoarthritis (OA) is a degenerative joint disease with high incidence in middle and old age, which the main pathological features are degenerative changes in the structure and function of articular cartilage [1, 2]. Chondrocytes are the only cells in articular cartilage tissue, which are distributed in the cartilage matrix and play an important role in maintaining the structural and functional integrity of articular cartilage [3]. Chondrocyte functional degeneration is one of the major contributing factors to the pathogenesis of OA [4]. Therefore, the regulation of chondrocyte proliferation, secretion, apoptosis, autophagy and other physiological functions is the key to the prevention and treatment of OA [5]. Under normal conditions, the proliferation and apoptosis of chondrocytes maintain a dynamic balance [6]. A large number of inflammatory factors contained in OA joint fluid can induce excessive proliferation of chondrocytes, destroy the homeostasis of articular cartilage, and aggravate cartilage degeneration [7].

At present, with the deepening of the study on the pathogenesis of OA, the regulation effect of long non-coding RNAs (lncRNAs) on the occurrence and development of OA has been continuously discovered [8, 9]. LncRNAs is a class of non-coding RNA molecules with a length of over 200 nucleotides, which is not only related to the growth, drug resistance and metastasis of tumors, but also involved in the synthesis and metabolism of cartilage matrix, proliferation and apoptosis of chondrocytes and other processes [10–12]. Antisense HOXA terminal transcriptional RNA (HOTTIP) is a non-coding RNA molecule derived from the 5′ -terminal of HOXA [13]. It has been found that lncRNA-HOTTIP is an oncogenic factor, which is involved in and promotes the development of liver cancer, gastric cancer, lung cancer, tongue squamous cell carcinoma and other tumors [14–16]. In addition, recent studies have found that lncRNA-HOTTIP may be involved in the occurrence and development of OA [17].

MicroRNA (miRNA) is a class of endogenous single-strand non-coding small molecule RNA with a length of about 18–22 nucleotides [18]. Studies have found that miRNA is not only involved in the physiological processes such as the growth, proliferation, differentiation and apoptosis of normal cells, but the abnormal expression of miRNA is also related to the occurrence and development of OA [19, 20]. MiRNA-663a is a miRNA that can promote or inhibit cancer in different tumors [21]. Studies have found that miRNA-663a can promote the progression of prostate cancer and lung cancer, and play a tumor suppressive role in gastric cancer [22, 23]. Fyn-related kinase (FRK) is a member of the Src kinase family and plays a role in promoting or inhibiting tumor growth in different types of tumors [24]. Studies have shown that FRK can promote the invasion of liver cancer and pancreatic cancer cells, and inhibit the metastasis of breast cancer and glioma cells [25–27]. However, there are no studies on the mechanism of miRNA-663a and FRK in the occurrence and development of OA. In this study, human OA articular cartilage and OA chondrocyte models induced by IL-1β were selected as subjects to explore the specific mechanism of lncRNA-HOTTIP, miRNA-663a and FRK in the progression of OA, so as to provide certain theoretical basis for the treatment of OA.

## Methods

### Tissues collection

Normal cartilage tissues were obtained from patients who were underwent the amputation without OA or rheumatoid arthritis history in Hospital of Fudan University (*n* = 30). In addition, OA patients who underwent total knee replacement (n = 30) were selected to obtain OA cartilage tissue. Articular cartilage tissues were preserved in a − 80 °C refrigerator. Because molecular properties can be different among each joint [28], we applied the box plot to evaluate the difference of HOTTIP expression between non-OA and OA samples. This study was approved by the ethics committee of Hospital of Fudan University. Each patient was notified and signed the written informed consent.

### Chondrocytes culture

First, the cartilage was digested using trypsin and collagenase II, and then the digested chondrocytes were separated. Chondrocytes were cultured using DMEM medium (10% FBS). The subsequent experiments used 10 ng/mL IL-1β to establish the OA chondrocyte model.

### The qRT-PCR assay

Total RNA was extracted from OA cartilage tissue or chondrocytes using Trizol reagent (Invitrogen, Carlsbad, USA). The extracted total RNA was reversely transcribed into cDNA. Next, SYBR Green PCR Kit (Takara, Japan) was used for real-time PCR amplification of primer fragments. GAPDH was used as the internal reference of mRNA and lncRNA, and U6 was used as the internal reference of miRNA. The relative expression level of each gene was calculated by 2^-△△Ct^ method.

### Cell transfection

Oligonucleotides or recombinant plasmids were transfected into the OA chondrocyte model using the Lipofectamine 3000 reagent (Life Technologies, Carlsbad, CA, USA). HOTTIP plasmid, miR-663a plasmid, FRK plasmid, si-HOTTIP, miR-663a-inh, si-FRK and their respective corresponding negative controls were purchased from Sangon Biotech (Shanghai, China).

### CCK8 assay

In this experiment, CCK-8 was used to determine the proliferation activity of OA chondrocytes, and the specific steps were strictly in accordance with the instructions of the kit. Simply put, the transfected OA chondrocytes were inoculated in a 96-well culture plate. At the end of the culture, the medium was replaced with fresh medium containing 10% CCK8. After incubation for 2 h, the absorbance value at 450 nm was determined by a microplate reader.

### Colony-forming analysis

The cells were treated with 0.3% soft AGAR and inoculated in 6-well plates (500 cells/Wells), and cultured in DMEM medium containing 10% FBS for 14 days. After the culture, the colony was fixed with methanol for 15 min, and then stained with 0.1% crystal violet for 15 min. After washing away, the excess crystal violet dye, the colony was photographed and counted.

### Detection of the cell apoptosis rate

The apoptosis rate of cells was detected using the membrane-linked protein V-FITC apoptosis assay kit (Invitrogen). Chondrocytes were digested by trypsin and re-suspended in the culture medium. The percentage of apoptosis was measured by flow cytometry after membrane protein V-FITC and PI dark staining.

### Double luciferase reporter gene assay

The Lipofectamine 3000 reagent was used to transfection the mutant HOTTIP (HOTTIP-MUT) or wild-type HOTTIP (HOTTIP-WT) vectors with miR-663a mimic or miR-NC into the OA chondrocyte model, and the interaction between HOTTIP and miR-663a was determined. In addition, the Lipofectamine 3000 reagent was used to transfection mutant FRK (FRK-MUT) or wild-type FRK (FRK-WT) vector with miR-663a mimic or miR-NC into OA chondrocytes, and the interaction between FRK and miR-663a was determined. After 48 h of transfection, luciferase activity in chondrocytes was determined using the kit (Promega, USA).

### RNA pull-down assay

The biotinylated HOTTIP was transfected into the OA chondrocyte model, and then the cells were lysed. The M-280 streptomyces anti-biotin magnetic beads (Invitrogen, USA) were co-incubated with the chondrocyte lysis products to form the biotin-miRNA-lncRNA complex. The immuno-precipitated RNA was purified by protease K and analyzed by qRT-PCR.

### Western blot assay

Total proteins in cells were extracted using radioimmunoprecipitation (RIPA) lysate and phenylmethanesulfonyl fluoride (PMSF), and quantified using bicinchoninic acid (BCA) protein concentration kit (Beyotime). Total protein was separated by 12% SDS-PAGE gel and transferred to cellulose acetate membrane. Then, 5% skim milk was used to close the membrane for 2 h. Next, the primary antibody was used to incubate the membrane under 4 °C for the night, and then the second antibody was used to incubate for 1 h at room temperature. Enhanced chemiluminescence (ECL) reagent (Beyotime) was used to color the protein bands, and GAPDH was used as the internal reference of the protein.

### Statistical analysis

All data are expressed as mean standard ± deviation (SD). T-test was used for significance analysis. *P* < 0.05 indicated a significant difference.

## Results

### Effect of HOTTIP on proliferation and apoptosis of IL-1β-induced chondrocytes

**Fig. 1:**
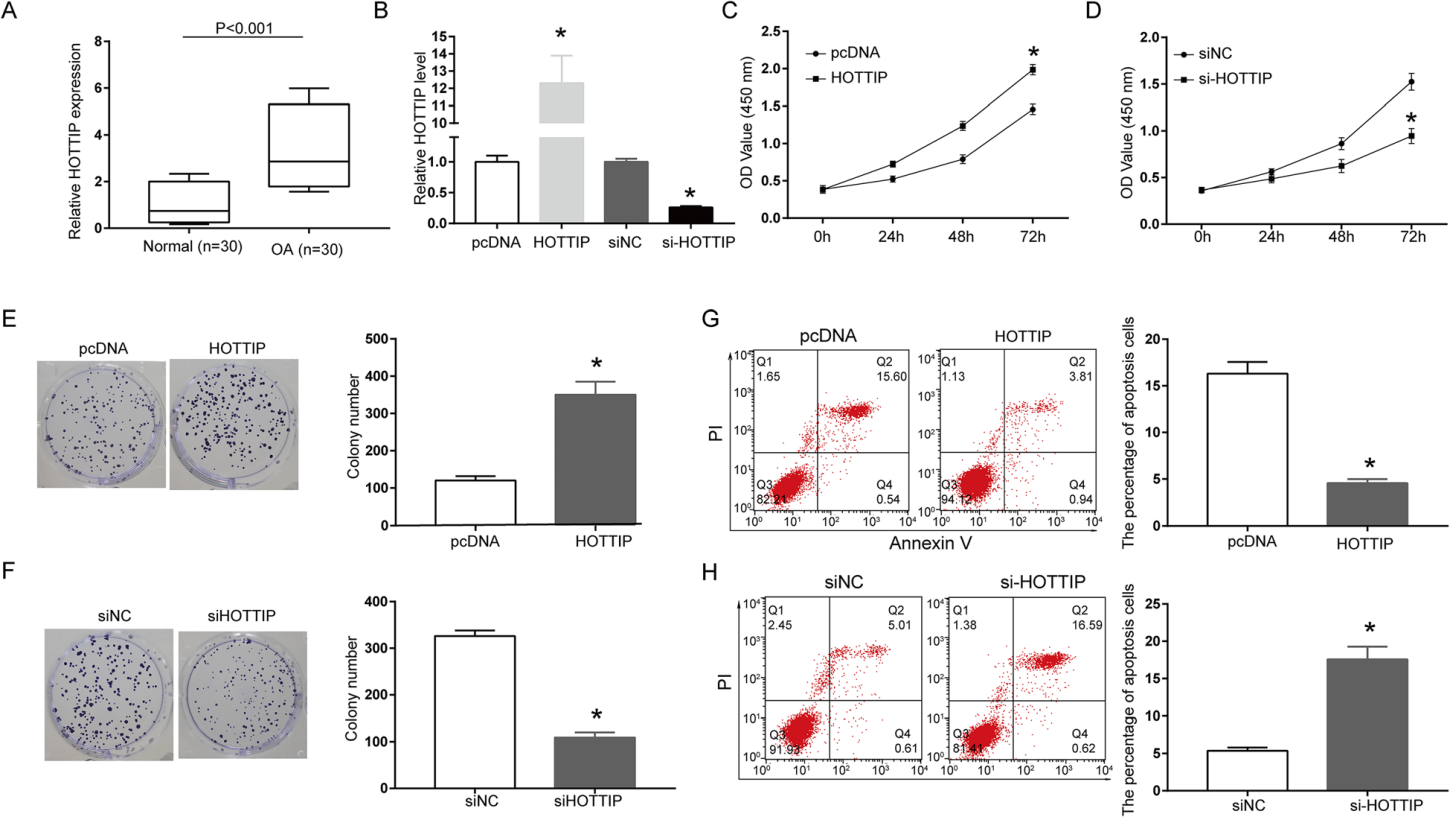
Effects of HOTTIP on proliferation and apoptosis of IL-1β-induced chondrocytes. **a** Determination of the expression of HOTTIP in human OA cartilage (*n* = 30) and normal cartilage (*n* = 30) by qRT-PCR. **b** Determination of the expression of HOTTIP in OA chondrocytes transfected with si-HOTTIP or pcDNA-HOTTIP. **c**, **d** Determination of the proliferation activity of OA chondrocytes transfected with pcDNA-HOTTIP (**c**) or si-HOTTIP (D) by CCK-8. **e**, **f** Determination of the colony formation ability of OA chondrocytes transfected with pcDNA-HOTTIP (**e**) or si-HOTTIP (**f**) by colony formation. **g**, **h** Determination of the percentage of apoptosis in OA chondrocytes transfected with pcDNA-HOTTIP (**g**) orsi-HOTTIP (**h**) by flow cytometry. *P < 0.05

The expression of HOTTIP in human OA cartilage (*n* = 30) and normal cartilage (*n* = 30) was determined by qRT-PCR. The results showed that the expression of HOTTIP in OA cartilage tissues was remarkably higher than that in normal cartilage tissues (Fig. 1a, *P* < 0.001), indicating that HOTTIP was highly expressed in OA cartilage tissues. In addition, IL-1β was used to induce the OA chondrocyte model, and si-HOTTIP or pcDNA-HOTTIP was transfected into OA chondrocytes to determine the effect of HOTTIP on the proliferation and apoptosis of OA chondrocytes. The results showed that the transfection of pcDNA-HOTTIP observably up-regulated the expression of HOTTIP, while the transfection of si-HOTTIP significantly down-regulated the expression of HOTTIP, suggesting that the transfections were successful. Furthermore, pcDNA-HOTTIP transfection remarkably enhanced the cell viability of the OA chondrocytes (Fig. 1c and e, *P* < 0.05), while si-HOTTIP transfection significantly inhibited the cell viability of the OA chondrocytes (Fig. 1d and f, *P* < 0.05). Moreover, flow cytometry results found that pcDNA-HOTTIP transfection remarkably reduced the apoptosis rate of OA chondrocytes, while si-HOTTIP transfection significantly increased the apoptosis rate of OA chondrocytes (Fig. 1g and h, *P* < 0.05). The above results indicated that HOTTIP was highly expressed in OA cartilage tissues, and the overexpressed HOTTIP could enhance the cell viability and inhibit the apoptosis of OA cartilage cells.

### Identification of miR-663a as the target gene of lncRNA-HOTTIP

**Fig. 2:**
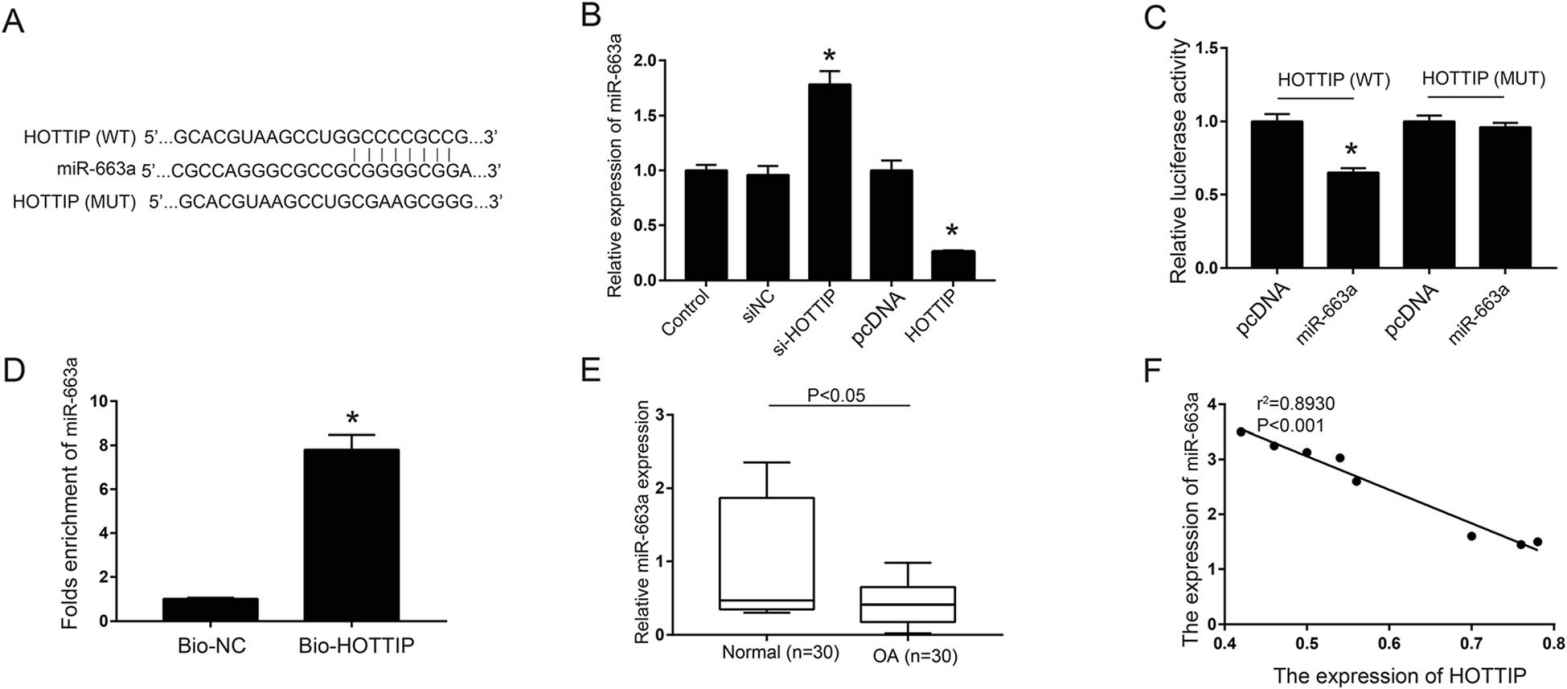
Identification of miR-663a as a target gene of lncRNA-HOTTIP. **a** Schematic diagram of predicted HOTTIP binding sites in miR-663a. **b** Determination of the expression of miR-663a in OA chondrocytes transfected with si-HOTTIP or pcDNA-HOTTIP. **c** Determination of luciferase activity in the OA chondrocyte model. **d** Determination of the relative enrichment of miR-663a in the OA chondrocyte model transfected with HOTTIP-Bio by RNA pull-down. **e** Determination of the expression of miR-663a in human OA cartilage (*n* = 30) and normal cartilage (*n* = 30) by qRT-PCR. **f** The correlation analysis of HOTTIP and miR-663a gene expression in OA cartilage tissue. * *P* < 0.05

The miRcode software was used to predict the possible targeted binding sites of HOTTIP in miR-663a (Fig. 2a). The qRT-PCR results found that pcDNA-HOTTIP transfection remarkably decreased the expression of miR-663a, while si-HOTTIP transfection observably increased the expression of miR-663a (Fig. 2b, *P* < 0.05). In addition, the HOTTIP-MUT or HOTTIP-WT vector was co-transfected with miR-663a mimic or miR-NC into the OA chondrocytes to determine the interaction between HOTTIP and miR-663a. The results showed that the luciferase activity of cells in the HOTTIP-WT group was significantly lower than that in the HOTTIP-MUT group after co-transfection with miR-663a mimics (Fig. 2c, *P* < 0.05). Furthermore, RNA pull down results showed that the relative enrichment of miR-663a in the OA chondrocytes transfected with HOTTIP-Bio was remarkably higher than that in the control group (Fig. 2d, *P* < 0.05). Moreover, we also determined the correlation between HOTTIP and miR-663a gene expression in OA cartilage tissues. The results found that miR-663a was significantly down-regulated in OA cartilage tissues compared with control group (Fig. 2e). Moreover, there was a significant negative correlation between miR-663a and HOTTIP gene expression in OA cartilage tissues (Fig. 2f, *P* < 0.001). The above results confirm that miR-663a is a direct target gene of HOTTIP in the OA chondrocytes.

### Effect of miR-663a on the proliferation and apoptosis of OA chondrocytes mediated by HOTTIP

**Fig. 3:**
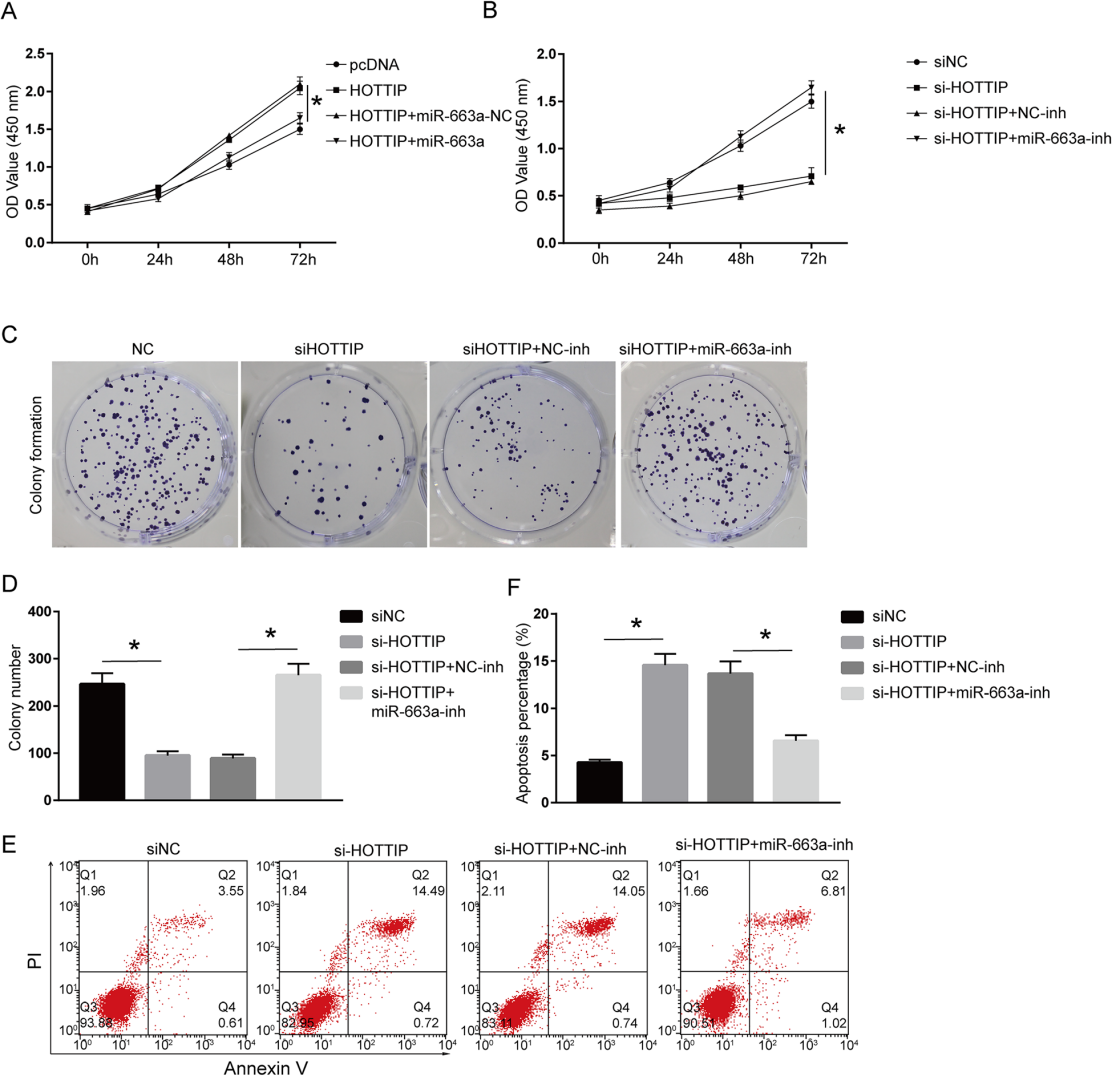
Effects of miR-663a on proliferation and apoptosis of OA chondrocyte model mediated by HOTTIP. **a** Determination of the cell viability of OA chondrocytes transfected with miR-663a mimic or/and pcDNA-HOTTIP by CCK-8. **b** Determination of the cell viability of OA chondrocytes transfected with miR-663a inhibitor or/and si-HOTTIP by CCK-8. **c**, **d** Determination of the colony formation ability of OA chondrocytes by colony formation. **e**, **f** Determination of the apoptosis percentage of OA chondrocytes by flow cytometry. *P < 0.05

We transfected the OA chondrocytes with miR-663a mimic or/and pcDNA-HOTTIP. It was found that miR-663a mimic significantly inhibited the cell viability enhanced by overexpressed HOTTIP (Fig. 3a, *P* < 0.05). In addition, we transfected the OA chondrocytes with miR-663a inhibitor or/and si-HOTTIP. The results showed that miR-663a inhibitor remarkably enhanced the cell proliferation reduced by HOTTIP knockdown (Fig. 3b-d, *P* < 0.05). In addition, flow cytometry showed that miR-663a inhibitor observably reduced the apoptosis induced by HOTTIP knockdown (Fig. 3e and f, *P* < 0.05). These results suggest that HOTTIP can competitively bind with miR-663a in OA chondrocytes, thereby improving cell proliferation and inhibiting apoptosis.

### Identification of FRK as the target gene of miR-663a

**Fig. 4:**
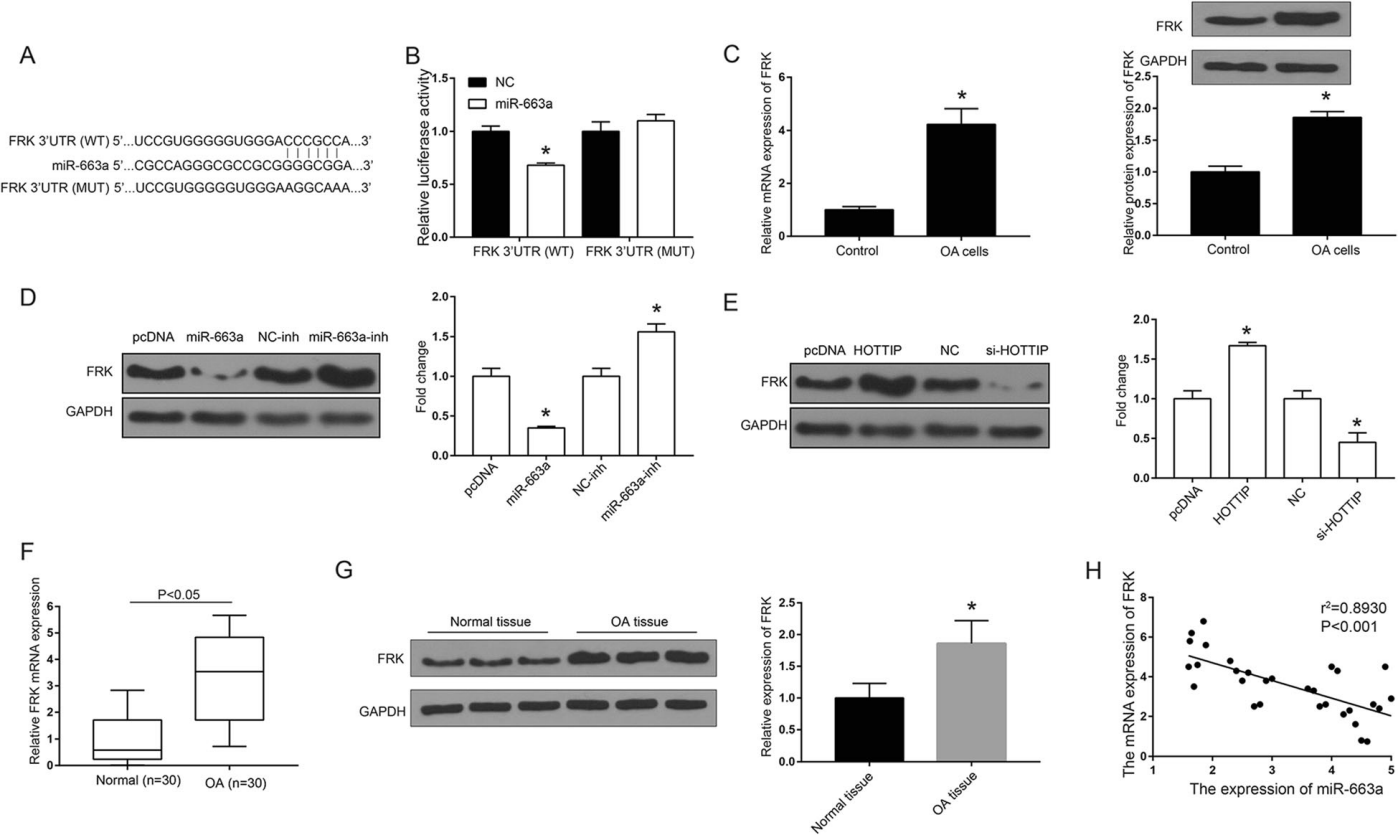
Identification of FRK as a target gene of miR-663a. **a** Schematic diagram of predicted binding sites of miR-663a in FRK. **b** Determination of luciferase activity in the OA chondrocytes. **c** The mRNA and protein expression of FRK in OA chondrocytes. **d** The effects of miR-663a on the the protein expression of FRK in OA chondrocytes. **e** The effects of HOTTIP on the the protein expression of FRK in OA chondrocytes. **f** The protein expression of FRK in OA cartilage tissues. **g** Determination of the expression of FRK in human OA cartilage (*n* = 30) and normal cartilage (*n* = 30) by qRT-PCR. **h** The correlation analysis of miR-663a and FRK gene expression in OA cartilage tissue. *P < 0.05

TargetScan was used to predict the potential binding sites of miR-663a in FRK (Fig. 4a). The results found that after co-transfection with miR-663a mimic, the luciferase activity of cells in FRK-WT group was remarkably lower than that in FRK-MUT group (Fig. 4b, *P* < 0.05). The mRNA and protein expression of FRK was found to be significantly up-regulated in OA chondrocytes compared with control cells (Fig. 4c, *P* < 0.05). Moreover, overexpression of miR-663a significantly down-regulated the protein expression of FRK in the OA chondrocytes, while inhibition of miR-663a observably up-regulated the protein expression of FRK in the OA chondrocytes (Fig. 4d, *P* < 0.05). Furthermore, overexpression of HOTTIP significantly up-regulated the protein expression of FRK in the OA chondrocytes, while knockdown of HOTTIP decreased the protein expression of FRK in the OA chondrocytes (Fig. 4e, *P* < 0.05). Besides, we found that the mRNA and protein expression of FRK was significantly up-regulated in OA cartilage tissue compared with control group (Fig. 4f and g). There was a significant negative correlation between FRK and miR-663a gene expression in OA cartilage tissue (Fig. 4h, *P* < 0.001). Taken together, HOTTIP up-regulated the expression of FRK by competitive binding with miR-663a.

### MiR-663a was involved in the proliferation and apoptosis of OA chondrocytes mediated by FRK

**Fig. 5:**
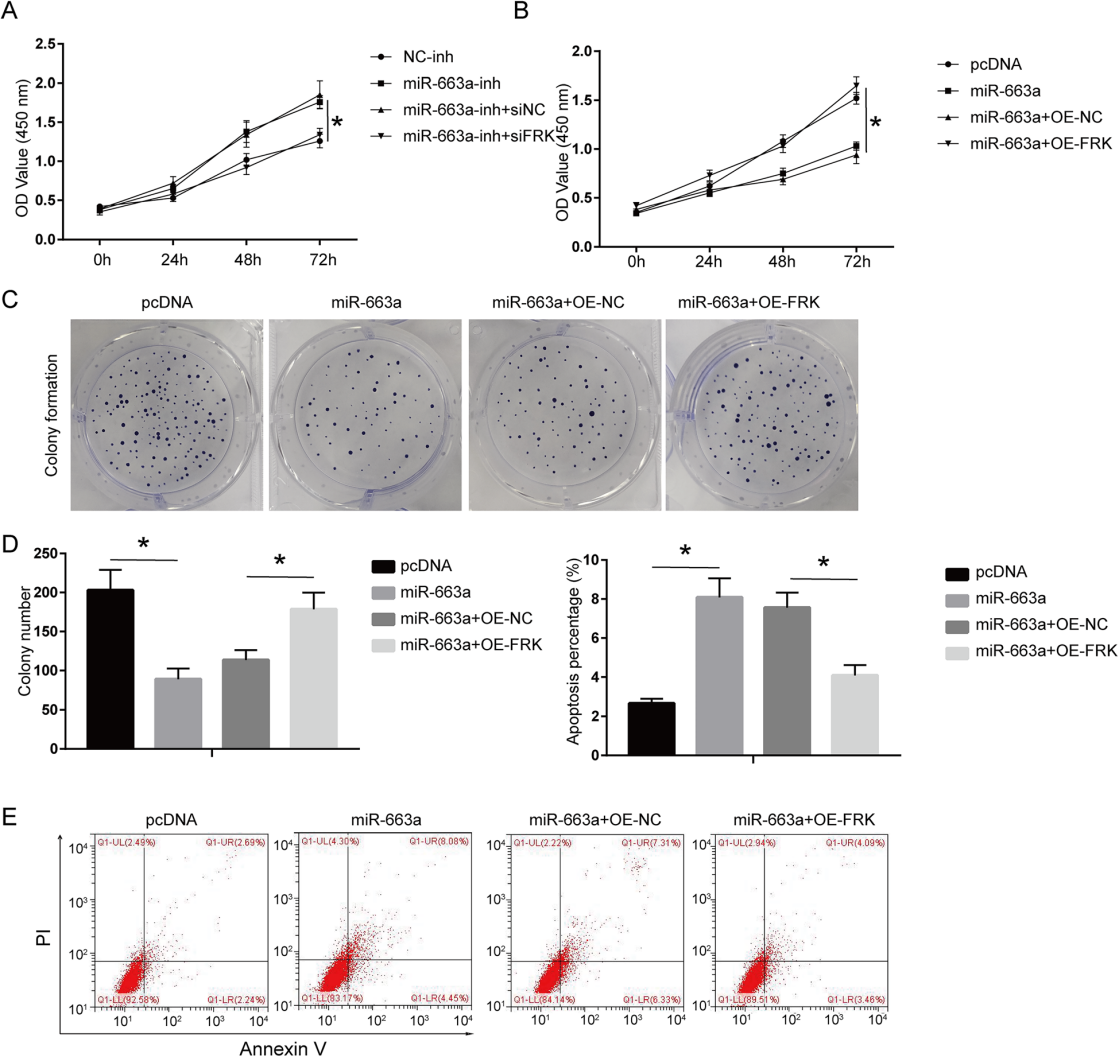
MiR-663a inhibits proliferation and apoptosis of OA chondrocyte model mediated by FRK. **a** Determination of the cell viability of OA chondrocytes transfected with miR-663a-inh or/and siFRK by CCK-8. **b** Determination of the cell viability of OA chondrocytes transfected with miR-663a mimic or/and OE-FRK by CCK-8. **c**, **d** Determination of the colony formation ability of OA chondrocytes by colony formation. **e** Determination of the apoptosis percentage of OA chondrocytes by flow cytometry. *P < 0.05

We transfected the OA chondrocyte model with miR-663a overexpression or/and miR-663a-inh. It was found that miR-663a mimic significantly inhibited the cell viability, and miR-663a-inh promoted cell proliferation (Fig. 5a and b, *P* < 0.05). Co-transfection results indicated that FRK overexpression could reverse the effect of miR-663a mimic on cell proliferation, while FRK knockdown reversed the effect of miR-663a-inh (Fig. 5a and b, *P* < 0.05). In addition, miR-663a inhibited the colony formation and reduced the apoptosis of OA chondrocytes, while this effect was reversed by overexpression of FRK (Fig. 5c-e, *P* < 0.05).

## Discussion

OA is a degenerative disease of the structure and function of articular cartilage, mainly occurring in middle-aged and elderly patients [29]. It has been found that HOTTIP not only promotes the development of liver cancer, gastric cancer, lung cancer, tongue squamous cell carcinoma and other tumors, but also participates in the occurrence and development of OA [17, 30, 31]. Kim et al. found that HOTTIP was highly expressed in OA articular cartilage, thereby inhibiting the synthesis of forward regulating protein integrin α1 in the process of endochondral osteogenesis, and ultimately accelerating the degeneration of articular cartilage [32]. In this study, we found that HOTTIP was highly expressed in OA cartilage tissues, which was consistent with the results reported in the literature [32]. Imbalance of chondrocyte proliferation and apoptosis is one of the main factors inducing OA, while lncRNAs are closely related to chondrocytes [11, 33]. Studies have showed that lncRNA HOX transcript antisense intergenic RNA (HOTAIR) can inhibit the apoptosis of chondrocytes [34], lncRNA Dnm3os can affect the proliferation and differentiation of chondrocytes [35], and lncRNA differentiation antagonizing non-protein coding RNA (DANCR) can promote the secretion of inflammatory factors in chondrocytes [36], thus participating in the process of OA progression. Currently, IL-1β has been widely used to establish an in vitro model of OA chondrocytes [37]. In the present study, the OA chondrocyte model was established by IL-1β induction, and it was found that overexpression of HOTTIP could enhance the cell viability of OA chondrocytes and inhibit apoptosis.

Studies have shown that lncRNA can bind to miRNA as competitive endogenous RNA (ceRNA), thus inhibiting the binding of miRNA to targeted mRNA [38]. MiRNA-663a is a miRNA involved in the occurrence and development of prostate cancer, lung cancer, gastric cancer and other cancers [39, 40]. The results of this study showed that there was a significant negative correlation between HOTTIP and the expression of miR-663a in OA cartilage tissues and OA chondrocytes, suggesting that HOTTIP might also play a biological role by competitively binding miR-663a. Therefore, this study predicted the existence of HOTTIP’s potential targeted binding sites in miR-663a through the biological information software miRcode. In addition, the double luciferase reporter gene and RNA pull down results confirmed that miR-663a was the direct target gene of HOTTIP in the OA chondrocyte model. This study further verified the effect of miR-663a on the proliferation and apoptosis of HOTTIP mediated OA chondrocyte model. The results showed that miR-663a mimic remarkably inhibited the cell viability enhanced by over-expressed HOTTIP, and miR-663a inhibitor significantly enhanced the cell viability inhibited by silent HOTTIP. Furthermore, flow cytometry assays showed that miR-663a inhibitor observably reduced the apoptosis induced by silencing HOTTIP. Zhang et al. found that lncRNA ubiquitinfold modifier conjugating enzyme 1 (UFC1) promoted the proliferation of OA chondrocytes and inhibited apoptosis by targeting miR-34a [41]. Thus, the results of this study suggested that HOTTIP could competitively bind with miR-663a in OA chondrocytes, thereby improving cell viability and inhibiting apoptosis.

Mature miRNAs can negatively regulate the expression of target genes at the transcriptional or translation level by binding to the 3’UTR region of the target genes, thus exerting their biological functions [42]. Yan et al. found that miRNA-34a could target regulating the sirtuin1 (SIRT1)/p53 signaling pathway, thereby affecting the proliferation and apoptosis of chondrocytes [43]. Wu et al. reported that miRNA-181 could inhibit the proliferation of OA chondrocytes and induce apoptosis by targeting phosphatase and tensin homolog (PTEN) [44]. As a member of the Src kinase family, FRK is involved in the progression of liver cancer, pancreatic cancer and breast cancer [27, 45]. In this study, there was a potential target binding site of miR-663a in FRK predicted by biological information software TargetScan. In addition, the double luciferase reporter assay confirmed the direct binding between FRK and miR-663a. Moreover, this study also found that FRK was remarkably negatively correlated with miR-663a expression in OA cartilage tissues and OA chondrocytes. The above results suggested that the overexpressed HOTTIP up-regulated the expression of FRK and ultimately promoted the occurrence and development of OA by competitive binding with miR-663a.

## Conclusion

In conclusion, this study revealed that HOTTIP played an important role in the cell proliferation and apoptosis of OA chondrocytes via miR-663a/ FRK axis. HOTTIP/miR-663a/FRK may be a potential target for the treatment of OA.

## Data Availability

The datasets used and/or analyzed during the current study are available from the corresponding author on reasonable request.

## Abbreviations

lncRNA: Long non-coding RNA
OA: Osteoarthritis
ceRNA: competitive endogenous RNA
HOTTIP: HOXA terminal transcriptional RNA
FRK: Fyn-related kinase
miRNA: Micro RNA
HOTTIP-MUT: HOTTIP
HOTTIP-WT: Wild-type HOTTIP
FRK-MUT: Mutant FRK
FRK-WT: Wild-type FRK
RIPA: Radioimmunoprecipitation
PMSF: Phenylmethanesulfonyl fluoride
BCA: Bicinchoninic acid
ECL: Enhanced chemiluminescence
HOTAIR: HOX transcript antisense intergenic RNA
DANCR: Differentiation antagonizing non-protein coding RNA
UFC1: Ubiquitinfold modifier conjugating enzyme 1
SIRT1: Sirtuin1
PTEN: Phosphatase and tensin homolog
